# Repeat catheter ablation vs. direct current cardioversion for recurrent atrial fibrillation after prior ablation: a propensity-matched analysis

**DOI:** 10.1093/europace/euag199

**Published:** 2026-08-31

**Authors:** Michel Abou Khalil, Mohammad Montaser Atasi, Yara Menassa, Maximilian Moersdorf, Christian M Massad, Alex El Darzi, Carlo El Khoury, Yishi Jia, Ghassan Bidaoui, Hadi Younes, Joe Abi-Rached, William Herbst, Charles Campbell, Eli Tsakiris, Abboud Hasan, Yingshuo Liu, Rodolfo Montiel Quintero, Omar Kreidieh, Amitabh C Pandey, Han Feng, Qussay Marashly, Nassir F Marrouche

**Affiliations:** Tulane Research Innovation for Arrhythmia Discovery, Tulane University School of Medicine, New Orleans, LA, USA; Tulane Research Innovation for Arrhythmia Discovery, Tulane University School of Medicine, New Orleans, LA, USA; Tulane Research Innovation for Arrhythmia Discovery, Tulane University School of Medicine, New Orleans, LA, USA; Tulane Research Innovation for Arrhythmia Discovery, Tulane University School of Medicine, New Orleans, LA, USA; Tulane Research Innovation for Arrhythmia Discovery, Tulane University School of Medicine, New Orleans, LA, USA; Tulane Research Innovation for Arrhythmia Discovery, Tulane University School of Medicine, New Orleans, LA, USA; Tulane Research Innovation for Arrhythmia Discovery, Tulane University School of Medicine, New Orleans, LA, USA; Tulane Research Innovation for Arrhythmia Discovery, Tulane University School of Medicine, New Orleans, LA, USA; Tulane Research Innovation for Arrhythmia Discovery, Tulane University School of Medicine, New Orleans, LA, USA; Tulane Research Innovation for Arrhythmia Discovery, Tulane University School of Medicine, New Orleans, LA, USA; Tulane Research Innovation for Arrhythmia Discovery, Tulane University School of Medicine, New Orleans, LA, USA; Tulane Research Innovation for Arrhythmia Discovery, Tulane University School of Medicine, New Orleans, LA, USA; Tulane Research Innovation for Arrhythmia Discovery, Tulane University School of Medicine, New Orleans, LA, USA; Tulane Research Innovation for Arrhythmia Discovery, Tulane University School of Medicine, New Orleans, LA, USA; Tulane Research Innovation for Arrhythmia Discovery, Tulane University School of Medicine, New Orleans, LA, USA; Tulane Research Innovation for Arrhythmia Discovery, Tulane University School of Medicine, New Orleans, LA, USA; Tulane Research Innovation for Arrhythmia Discovery, Tulane University School of Medicine, New Orleans, LA, USA; Tulane Research Innovation for Arrhythmia Discovery, Tulane University School of Medicine, New Orleans, LA, USA; Tulane Research Innovation for Arrhythmia Discovery, Tulane University School of Medicine, New Orleans, LA, USA; Tulane Research Innovation for Arrhythmia Discovery, Tulane University School of Medicine, New Orleans, LA, USA; Tulane Research Innovation for Arrhythmia Discovery, Tulane University School of Medicine, New Orleans, LA, USA; Tulane Research Innovation for Arrhythmia Discovery, Tulane University School of Medicine, New Orleans, LA, USA

Recurrent atrial fibrillation (AF) following catheter ablation occurs in 20–50% of patients within 5 years, predominantly due to pulmonary vein (PV) reconnection or non-PV triggers.^1^ Clinicians managing post-ablation recurrence face a practical dilemma: repeat catheter ablation, which targets the underlying electrophysiologic substrate, vs. direct current cardioversion (DCCV), a simpler but substrate-agnostic intervention. Prior studies have evaluated outcomes of ablation and cardioversion in anticoagulated AF patients^2^ and demonstrated the feasibility of blinded randomized comparisons of pulmonary vein isolation vs. DCCV in persistent AF,^3^ yet neither addressed the specific scenario of recurrence after a prior ablation. We therefore conducted a propensity-matched retrospective analysis to compare long-term clinical outcomes of repeat ablation vs. DCCV in this population.

We conducted a retrospective cohort study using the TriNetX Research Network, a federated de-identified EHR platform (112 US healthcare organizations). Adults (≥18 years) with AF undergoing initial catheter ablation between 1 January 2015 and 1 January 2023 were identified. The Repeat Ablation cohort comprised patients who underwent a second ablation 1 month to 1 year after the index procedure, with no DCCV after the initial ablation. The DCCV cohort comprised patients who underwent electrical cardioversion within the same window, without repeat ablation. The index event was the qualifying procedure; outcomes were tracked over 2 years.

The primary endpoint was a composite of all-cause mortality, ischaemic or haemorrhagic stroke and acute heart failure within 730 days. Secondary endpoints were each component individually, including ischaemic stroke alone. Propensity score matching 1:1 was performed on 66 covariates: demographics (age, sex, race, body mass index); comorbidities (hypertension, heart failure subtypes, ischaemic heart disease, diabetes, chronic kidney disease, prior stroke, and AF subtype); medications (β-blockers, angiotensin-converting enzyme inhibitors, angiotensin II receptor blockers, diuretics, anticoagulants, and antiarrhythmics including amiodarone, flecainide, dofetilide, sotalol, and dronedarone); and laboratory values (creatinine, LDL, triglycerides, hemoglobin A1c, B-type natriuretic peptide, left ventricular ejection fraction (LVEF)). The Kaplan–Meier and Cox proportional hazards analyses were performed within the TriNetX platform (R v4.0.2).

Before matching, 2227 patients underwent repeat ablation and 5009 underwent DCCV. Unmatched cohorts differed substantially: DCCV patients were older (67.5 ± 9.9 vs. 63.2 ± 10.7 years), had higher rates of persistent AF (80.8% vs. 52.2%), heart failure (46.9% vs. 26.1%), and greater diuretic use (73.8% vs. 50.9%). After propensity score matching, 1569 patients per group were retained with well-balanced baseline characteristics (all standardized mean differences <0.1). Both cohorts had a mean age of 65 years, were ∼67% male and ∼87% white, with a mean LVEF of ∼54%, baseline heart failure in ∼33%, and comparable antiarrhythmic drug use (amiodarone ∼35%, flecainide ∼23%, dofetilide ∼15%, sotalol ∼15%). Oral anticoagulation use was balanced (apixaban ∼59%, rivaroxaban ∼31%). Persistent AF was present in ∼57% of each matched cohort.

The primary composite outcome occurred in 205 patients (13.1%) with repeat ablation vs. 314 (20.0%) with DCCV [hazard ratio (HR) 0.65; 95% confidence interval (CI) 0.54–0.77; *P* < 0.001; number needed to treat (NNT) = 14, log-rank *P* < 0.001; *Figure 1A*]. All-cause mortality was 2.5% vs. 5.5% (HR 0.47; 95% CI 0.32–0.68; *P* < 0.001; NNT = 33; *Figure 1B*). Total stroke occurred in 3.5% vs. 5.3% (HR 0.67; 95% CI 0.48–0.95; *P* = 0.023; NNT = 56; *Figure 1C*); ischaemic stroke alone was 3.2% vs. 4.8% (HR 0.68; 95% CI 0.47–0.97; *P* = 0.032; NNT = 63). Acute heart failure occurred in 8.7% vs. 13.1% (HR 0.66; 95% CI 0.53–0.82; *P* < 0.001; NNT = 23; *Figure 1D*).

**Figure 1:**
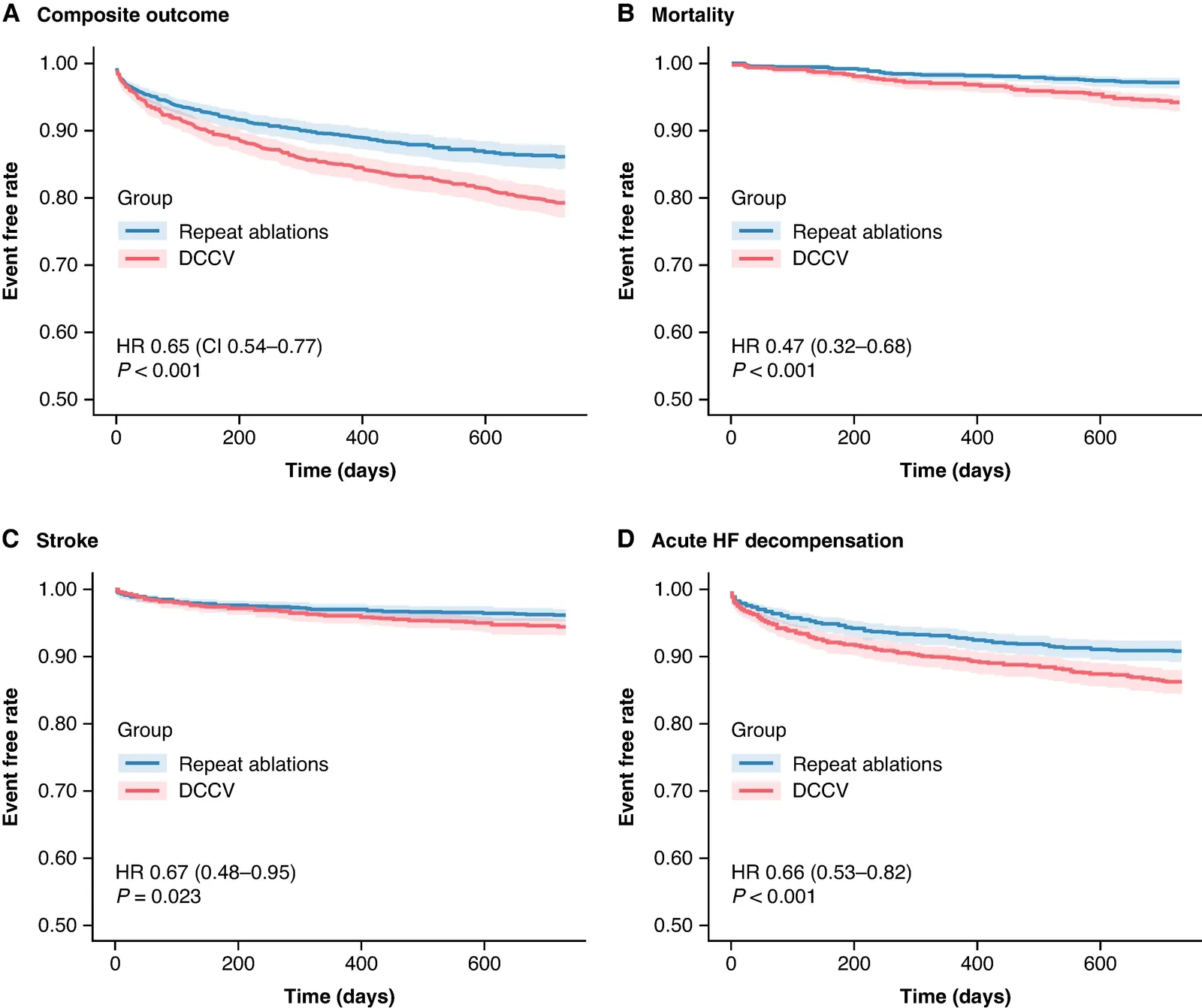
The Kaplan–Meier survival curves after repeat ablation vs. cardioversion. Four-panel Kaplan–Meier analysis comparing repeat catheter ablation and direct current cardioversion over 2 years of follow-up. (*A*) Composite outcome of all-cause mortality, stroke, or acute heart failure (HR 0.65; 95% CI 0.54–0.77; *P* < 0.001]. (*B*) All-cause mortality (HR 0.47; 95% CI 0.32–0.68; *P* < 0.001). (*C*) Ischaemic or haemorrhagic stroke (HR 0.67; 95% CI 0.48–0.95; *P* = 0.023). (*D*) Acute heart failure (HR 0.66; 95% CI 0.53–0.82; *P* < 0.001). Shaded areas represent 95% CIs. Numbers at risk are shown below each panel. AF, atrial fibrillation; CI, confidence interval; DCCV, direct current cardioversion; HF, heart failure; HR, hazard ratio.

To our knowledge, this is the first real-world study to directly compare hard clinical outcomes between repeat ablation and DCCV in patients with AF recurrence after prior ablation. In this large propensity-matched cohort, repeat ablation was associated with a 35% relative reduction in the composite endpoint (NNT = 14), with consistent benefit across mortality, stroke, and heart failure. Notably, ischaemic stroke alone was significantly lower with repeat ablation (3.2% vs. 4.8%; HR 0.68; *P* = 0.032), despite both groups being well matched for anticoagulation use, suggesting that residual stroke risk reflects ongoing arrhythmia burden rather than anticoagulation differences.

These findings align with evidence that DCCV restores sinus rhythm transiently without modifying atrial substrate, leading to frequent relapse and cumulative arrhythmia-related myocardial stress,^4^ whereas repeat ablation targets PV reconnection to achieve more durable rhythm control.^5^ They complement EAST-AFNET 4,^6^ which demonstrated that early rhythm control reduces cardiovascular events in newly diagnosed AF, and CASTLE-AF,^7^ which showed ablation superiority over medical therapy in AF with heart failure. In the CHINA-AF registry, repeat ablation was associated with a lower incidence of cardiovascular death, stroke, or major bleeding vs. medical therapy alone in patients with post-ablation recurrence (adjusted HR 0.56 at 3 years), further supporting durable rhythm control as a prognostically meaningful strategy.^8^ A randomized trial of persistent AF further confirmed markedly lower 12-month AF burden with ablation vs. DCCV-based strategies (17 ± 38% vs. 62 ± 49%; *P* < 0.0001).^9^

In the unmatched population, DCCV was performed more than twice as frequently as repeat ablation (5009 vs. 2227), reflecting a common practice of attempting one or serial cardioversions before re-ablation. Consistent with a recent study showing that repeat ablation within ∼7.5 months of recurrence significantly reduces second recurrence rates,^10^ earlier referral for re-ablation may prevent progressive atrial remodelling and improve long-term outcomes.

This study has important limitations. As a retrospective analysis of administrative data, causality cannot be inferred despite rigorous matching. Procedural details, PV reconnection rates, and antiarrhythmic co-administration at cardioversion were unavailable. Long-term rhythm monitoring data are absent, precluding assessment of post-procedure arrhythmia burden. The cohort is predominantly white (∼87%), limiting generalizability. Residual confounding from selection of healthier patients towards re-ablation cannot be excluded. Prospective randomized trials are needed to confirm these findings.

In conclusion, in a large propensity-matched real-world cohort, repeat catheter ablation was associated with significantly lower risks of all-cause mortality, stroke, and acute heart failure compared with DCCV in patients with recurrent AF after prior ablation. These findings support earlier consideration of repeat ablation as the preferred rhythm-control strategy in this population.

## Data Availability

The data that support the findings of this study are derived from the TriNetX Research Network (https://trinetx.com), a federated platform accessible to registered institutional users. De-identified aggregate data are available through the TriNetX platform subject to institutional data use agreements. Individual-level data cannot be shared publicly due to data governance restrictions.
